# Characterization of Self-Assembled 2D Patterns with Voronoi Entropy

**DOI:** 10.3390/e20120956

**Published:** 2018-12-11

**Authors:** Edward Bormashenko, Mark Frenkel, Alla Vilk, Irina Legchenkova, Alexander A. Fedorets, Nurken E. Aktaev, Leonid A. Dombrovsky, Michael Nosonovsky

**Affiliations:** 1 Department of Chemical Engineering, Biotechnology and Materials, Engineering Sciences Faculty, Ariel University, Ariel 407000, Israel; 2 University of Tyumen, 6 Volodarskogo St., Tyumen 625003, Russia; 3 Joint Institute for High Temperatures, 17A Krasnokazarmennaya St., Moscow 111116, Russia; 4 Mechanical Engineering, University of Wisconsin—Milwaukee, 3200 North Cramer St., Milwaukee, WI 53211, USA

**Keywords:** Voronoi entropy, surface patterns, Lewis law, Aboav law, droplet cluster, self-assembly

## Abstract

The Voronoi entropy is a mathematical tool for quantitative characterization of the orderliness of points distributed on a surface. The tool is useful to study various surface self-assembly processes. We provide the historical background, from Kepler and Descartes to our days, and discuss topological properties of the Voronoi tessellation, upon which the entropy concept is based, and its scaling properties, known as the Lewis and Aboav–Weaire laws. The Voronoi entropy has been successfully applied to recently discovered self-assembled structures, such as patterned microporous polymer surfaces obtained by the breath figure method and levitating ordered water microdroplet clusters.

## 1. Introduction

**Figure 1:**
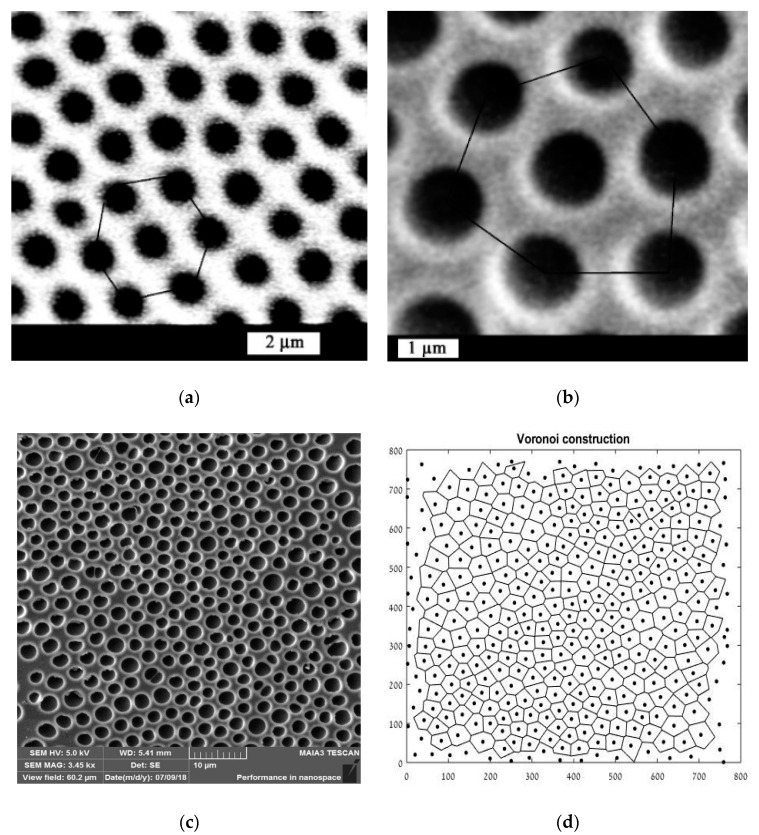
Porous ordered polycarbonate honeycomb structures obtained with breath-figures self-assembly is shown. (**a**) Scale bar is 2 µm. (**b**) Scale bar is 1 µm. (**c**) Scale bar is 10 µm. (**d**) Voronoi diagram for the case (c), S_vor_ = 1.0131, is depicted.

Many scientific and technological problems involve patterns with a surface distribution of spots. A common example is microscaled porous honeycomb patterns on a polymer’s surface arising from the so-called breath-figures self-assembly, which will be described in detail below [1,2,3,4] (Figure 1). Intuitively, the images of the pores in Figure 1a,b look ordered, whereas the pattern presented in Figure 1c seems to be disordered. But how this intuitive feeling can be quantified? Quantitative parameters of self-organization can be obtained by building the Voronoi diagram (also called the Voronoi tessellation, or Voronoi partition) and calculating the appropriate Voronoi entropy, which is the topic of the present paper [5]. An example corresponding to the case in Figure 1c is presented in Figure 1d.

**Figure 2:**
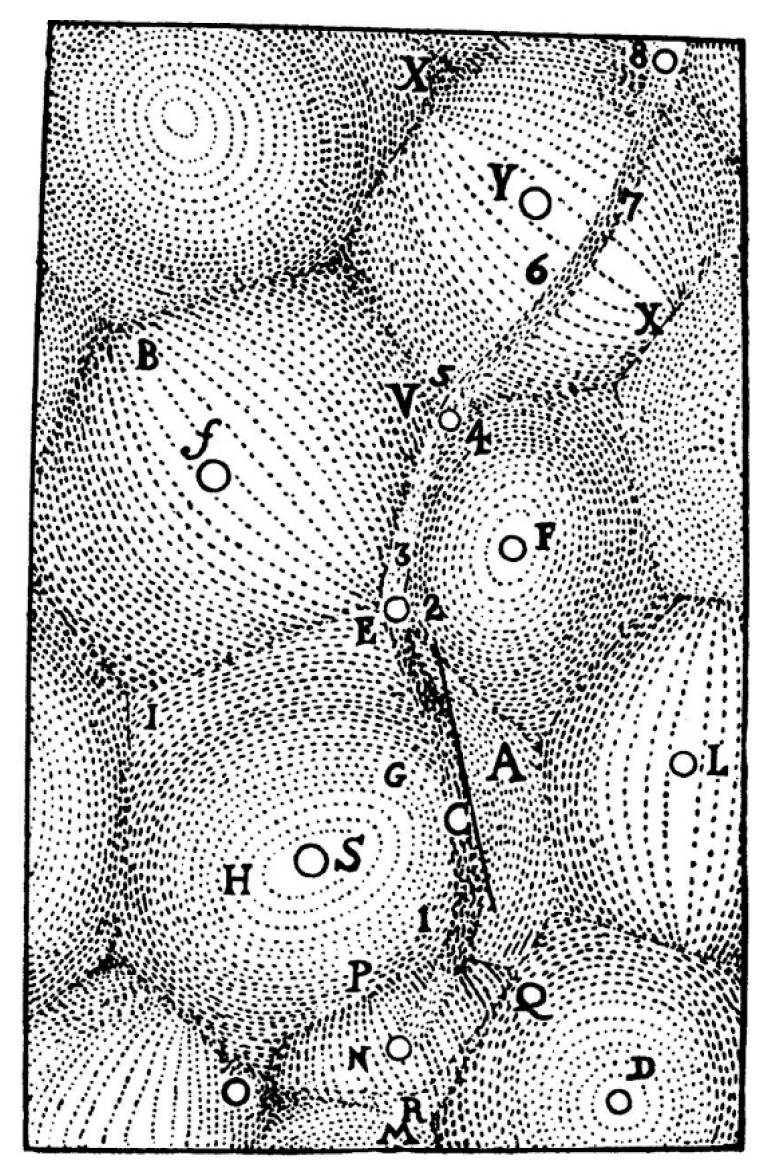
The tessellation diagram drawn by René Descartes is shown in [6,7]. Letters denote masses.

It appears that the idea of what is now called the Voronoi tessellation has been proposed already by Johannes Kepler and Rene Descartes in the 17th century [6,7]. Kepler used it to study the densest sphere packing problem, whereas Descartes employed these tessellations to verify that the distribution of matter in the Universe forms vortices centered at fixed stars (Figure 2) [6,7]. British physician John Snow, referred to as “the father of modern epidemiology,” re-discovered the tessellations during the 1854 London cholera outbreak [7,8]. Snow identified infected wells by superposing the map of cholera cases and the Voronoi diagram of the water sources sites [7,8], thus proving that Voronoi diagrams can even save lives. In parallel, the idea was revived by Dirichlet in the context of his works on quadratic forms [9].

Georgy Voronoi (1868–1908) was a student of Markov in Saint Petersburg University, who spent most of his career at the University of Warsaw where he had become a professor even before completing his PhD thesis [7]. Voronoi’s results were published in 1908, the year of his untimely death at the age of 40 [5].

**Figure 3:**
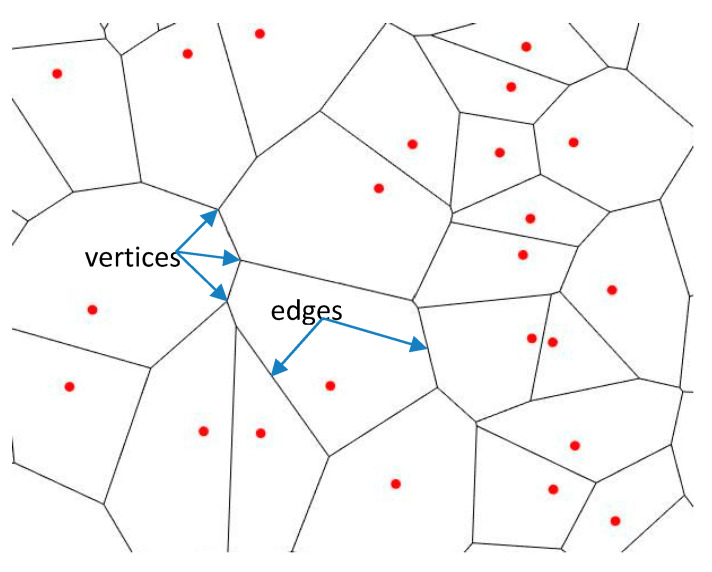
Example of the Voronoi tessellation on a set of points. Red points represent *seeds* or *nuclei.*

A Voronoi tessellation or diagram of an infinite plane is a partitioning of the plane into regions based on the distance to a specified discrete set of points (called *seeds, sites, nuclei*, or *generators*) [10,11]. For each seed, there is a corresponding region consisting of all points closer to that seed than to any other. The Voronoi polyhedron of a point nucleus in space is the smallest polyhedron formed by the perpendicularly bisecting planes between a given nucleus and all the other nuclei. The Voronoi tessellation divides a region into space-filling, non-overlapping convex polyhedral, shown in Figure 3 [10,11]. 

The Voronoi entropy calculated from the diagrams is used to quantify orderliness of sets of spots on a 2D plane or cells around these points. Such random or self-organized cells appear during various processes in the materials science and surface science including grain growth and self-assembly of colloidal and droplet patterns.

## 2. Topological and Scaling Properties of Voronoi Diagrams and Entropy

A Voronoi diagram has the following two salient properties: (i) the *edges* of the Voronoi diagram include all the points in the plane that are equidistant to the nearest seed, and (ii) the *vertices* are the points equidistant to three (or more) seeds. Topologically, Voronoi diagrams represent planar graphs with a number of interesting properties [12]. The number of edges joined to a given vortex is its *coordination number z*. A topologically stable Voronoi diagram, i.e., a diagram which maintains its topological properties under small deformations, is characterized by the coordination number of all its vortices z=3 [12]. Note that the Voronoi diagram, as any other planar cellular pattern, obeys the Euler equation (see Figure 3)

(1) −n+f=χ=2

where and *v* is the number of vertices, *n* is the number of edges, *f* is the number of cells (polygons bounded by edges including the outer infinitely large region) and χ is the Euler number (or the Euler characteristics) [12]. Consider that one of these cells is unbounded, and is called the infinite cell.

An immediate consequence of the Euler equation for the Voronoi diagrams is that in the limit of a large system (when *v*, *n*, and *f* are all large integers), the average number of edges surrounding a cell is six, or 〈n/f〉=3. This is because for topologically stable diagrams $n=\frac{3}{2}v$ (because three edges meet at every vertex and an edge links two vertices), which yields f=n/3 (see Reference [12]). 

The seeds sharing a common Voronoi segment are geometric neighbors [10,13]. When such common physico-chemical processes as the heterogeneous condensation or grain growth are considered, geometric neighbors become competing centers in a growth scenario.

To quantify the orderliness of the Voronoi tessellation or a similar 2D structure, the so-called Voronoi entropy is defined as

(2) $$S_{vor}=-\underset{n}{\sum }P_{n}\ln P_{n}$$

where *P_n_* is the fraction of polygons with *n* sides or edges (also called the coordination number of the polygon) in a given Voronoi diagram [10,11,12]. The summation in Equation (2) is performed from *n* = 3 to the largest coordination number of any available polygon, e.g., to *n* = 6 if a polygon with the largest number of edges is a hexagon. 

The Voronoi entropy can be viewed as a measure of information content in the diagram. The Voronoi entropy becomes zero for a perfectly ordered structure consisting of a single type of polygons, so that *P_n_* = 1 and ln *P_n_* = 0. For a typical case of a fully random 2D distribution of points (i.e., with a uniform probability distribution of seed points on a plane), the value of $S_{vor}=1.71$ has been reported [14]. Therefore, it is expected that for a self-organizing structure, the value of *S_vor_* decreases. Note that the Voronoi entropy is an intensive property, unlike the thermodynamic entropy, which is an extensive property. Therefore, the value does not depend on the number of seeds, which makes it appropriate to study processes where the number of seeds increases. 

The degree of randomness in a cellular structure with straight edges can be characterized by Lewis’ law [15,16,17,18,19,20,21]. Lewis observed a linear relationship between the average area of a typical *n*-polygon, $〈A_{n}〉$, and *n* for various random 2D cellular mosaics created by growing living cells at various stages of the development

(3) $$〈A_{n}〉=\alpha \left(n-2\right)$$

where *α* is a proportionality constant. Equation (3) suggests that the pattern can be considered random if there is a linear relationship between the number of edges and the mean area. For the precise value and meaning of the constant *α,* see Reference [19]. The validity of Lewis’ law was tested on natural patterns of different nature at different scale sizes, from micrometers to kilometers [22,23]. In particular, the Lewis scaling law was observed for patterns arising from condensation of droplets, which is crucial for the formation of the breath-figures patterns and condensed droplet clusters [4,23,24,25,26,27]. Another scaling law, which has also been suggested, is the Desch law stating a linear relation between the perimeter of polygons and the number of their edges [22,28]. 

Besides the Lewis and Desch laws, there is another important scaling law, related to Voronoi diagrams, which is called the Aboav law [29,30,31]. This law relates the average number of sides $m_{n}$ of a Voronoi cell that neighbors an *n*-sided cell to the number *n* according to:

(4) $$m_{n}=a+\frac{b}{n}$$

where *a* and *b* are constants. The Aboav law is often called in the literature the Aboav–Weaire law [31].

Hence, small grains tend to be surrounded by large ones and vice versa (more accurately speaking. the few-edged cells have a remarkable tendency to be in contact with many-edged cells and vice versa) [19,29,30]. The explanation of the Aboav law, exploiting the Euler formula (Equation (1)) was suggested, and the values of constants *a* and *b* appearing in Equation (4) were discussed in the literature [19,30].

Weaire in Reference [30] stated that the Aboav formula appears to derive inexorably from the 2D geometry and topology, and that it should not be seen as a departure from randomness [30]. 

Some other properties of random planar distributions of nuclei generating Voronoi diagrams are known. When the points are randomly and uniformly distributed on the plane, the probability *p_n_* that a point has a *n*-sided Voronoi cell is given, for large *n,* by

(5) $$p_{n}=\frac{const}{4\pi^{2}}\frac{{\left(8\pi^{2}\right)}^{n}}{\left(2n\right)!}\left[1+O\left(\frac{1}{\sqrt{n}}\right)\right]$$

which behaves as $p_{n}\approx n^{-2n}$. The area distribution of Voronoi cells for random patterns was suggested for the normalized cell size distribution function

(6) $$f\left(x\right)=const\times x^{\frac{3d-1}{2}}\exp\left(-\frac{\left(3d+1\right)x}{2}\right)$$

where *d* is the dimensionality of the space (d=1, 2, 3) [32]. The statistical distribution of perimeters of Voronoi cells inherent for random patterns was treated in Reference [33]. Recursive Voronoi diagrams created on a set of points can generate fractal patterns [34]. From the geometrical point of view, the Voronoi tessellation represents a dual graph of the Delaunay triangulation [35].

Multidimensional generalizations of the Voronoi diagrams are discussed in References [13,32]. The 3D Voronoi diagrams are used in crystallography. The Voronoi partition goes further than traditional crystallo-chemical models based on the spherical atoms, since they can include the effect of the crystal field on the atom shape. This introduces new methods of crystal structure description at the local and global levels, such as sphericity and uniformity criteria, topological parameters for atomic packings, and ionic arrays and methods for void subspace analysis. Voronoi partition turns out be useful for the quantitative analysis of the structure of void space in polymer solutions [36] and solid polymers [37].

## 3. Analysis of 2D Self-Assembled Surface Patterns with 2D Voronoi Diagrams 

Given that Voronoi diagrams can characterize ordering in diverse surface patterns, from random to regular, they are used to study self-assembled structures. Among the examples are kinetically driven self-assembly of highly ordered nanoparticle monolayers, formed by evaporation of colloidal solutions [38], 2D arrays of Au nanoparticles synthesized from a near-perfect hexagonal layer of diblock copolymer micelles by solvent vapor treatment [39], and epitaxial self-assembled nanostructures [40], Voronoi diagrams indicate the location of defected sites in self-assembled patterns, thus enabling immediate revealing of dislocations and defected areas [41].

Interestingly, Voronoi diagrams may arise in a natural way from self-assembly processes. Zambo et al. reported self-assembly of like-charged nanoparticles into Voronoi diagrams [42]. A macroscopic pattern was generated by the spatiotemporally controlled aggregation of like-charged carboxyl-terminated gold nanoparticles in a hydrogel, where clustering has been induced by the screening effect of the sodium ions that diffuse in a hydrogel [42]. Diffusion fronts of the sodium ions induced nanoparticle aggregations, which generated Voronoi structures, where the Voronoi cells consisted of aggregated nanoparticles and their edges represented aggregation-free and nanoparticle-free zones [43].

Martin et al. studied pattern formation during 2D nanoparticle self-assembly controlled by direct modification of solvent dewetting dynamics [43]. The authors compared three different techniques for the study of ordering in the resulting patterns: the Voronoi diagrams, two-dimensional fast Fourier transform analysis of the images [44], and the Minkowski functional method [45,46]. The Minkowski functionals of point patterns are calculated by centering a disk on each point and analyzing the topology of this secondary patterns of overlapping disks as a function of the radius [45]. 

By combining the overlapping disks, a pattern of differently shaped objects is formed. The total area of this collection of objects is then just the total area of the disks excluding any overlapping area. This is the first Minkowski measure (functional). The second Minkowski measure, the total perimeter of the pattern, is the perimeter of all of the shapes, which is reduced from the perimeter of the individual disks because of overlaps. The Euler number *χ*, supplied by Equation (1) is the final Minkowski measure, defined as the total number of distinct shapes or components in the window (created by the overlapping disks) minus the number of holes [45]. Mathematically, the three functionals do completely classify a pattern [45]. It was suggested that the Minkowski functional method is the most comprehensive for the recognition of inherent ordering for point patterns [45]. A comparison of the effectiveness of the Fourier transform, Minkowski functionals, and Voronoi diagrams for characterization of ordering in point patterns still remains an open problem.

**Figure 4:**
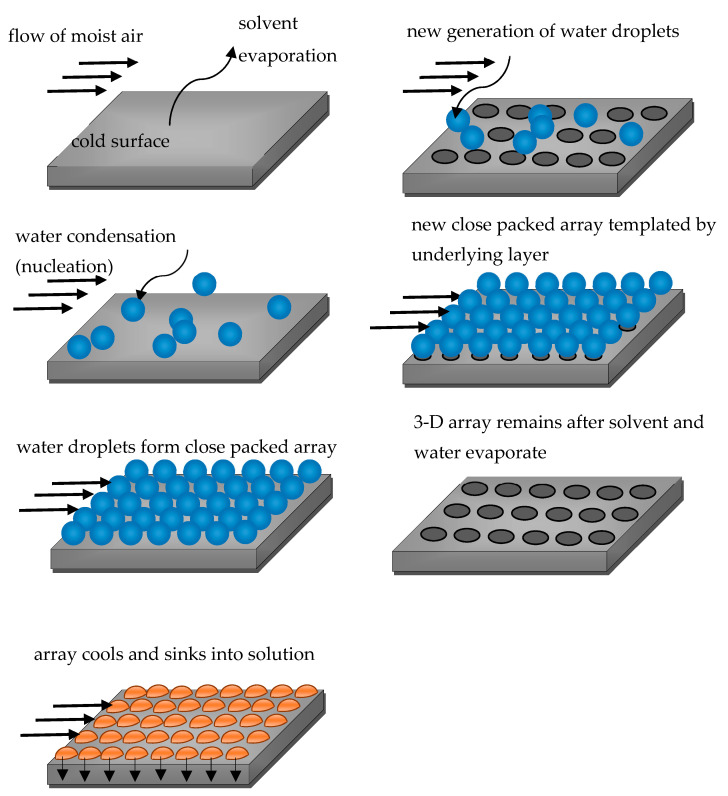
The main stages of the breath figures self-assembly, resulting in creation of ordered honeycomb microporous topographies, are depicted.

Another method enabling characterization of patterning in 2D self-assembled patterns with the correlation functions was reported in Reference [47], in which porous honeycomb structures arising from breath figure self-assembly [1,2,3,4,14,25,26,27], depicted schematically in Figure 4, were studied.

These patterns are formed by the so-called breath figure self-assembly process. The breath figures refer to the fog that forms when water vapor contacts a cold, typically solid surface, such as glass. The common example is the fog which appears on a window, when one breathes on it. The formation of breath figures was first studied more than hundred years ago by J. Aiken and by Lord Rayleigh [48,49,50,51]. Breath figures can form highly regular hexagonal arrangements of fog microdroplets. This is apparently due to their non-coalescence and due to various interactions (such as the Marangoni convection) and variations in the temperature and humidity next to condensing microdroplets [4]. 

In the 1990s, it was discovered that breath figures can play a significant role in materials science due to formation of regular honeycomb arrangements of micropores on the surface of polymers, formed by rapid evaporation of polymer solutions in humid atmosphere. Rapid evaporation of the solvent cools the solution–humid air interface, resulting in intensive condensation of water droplets at the interface. The droplets then sink into the solution, eventually forming a honeycomb pattern (Figure 4). These breath figure patterns are used to synthesize superhydrophobic surfaces [4].

Scanning electron microscopy (SEM) images of breath-figures patterns were treated as follows: in order to understand the short-range and long-range ordering in the obtained 2D structures, the statistical properties of the auto-correlation functions were analyzed [47]. The correlational analysis of the SEM images indicated short-range (ca. 5 µm) and large-scale (ca. 50 µm) ordering of the honeycomb structures [47]. There is limited research addressing the Voronoi-partition-based analysis of hierarchical 2D patterns [43,52,53]. A generalized version of the Voronoi-Delaunay method was used to study relatively large intermolecular voids [54]. The suggested version made the Voronoi diagrams applicable for molecular systems, i.e., ensembles of partly overlapping spheres [50]. 

Regrettably, the majority of studies reporting application of Voronoi diagrams to the study of synthetic self-assembled patterns did not concentrate on the validity of the Lewis [15,16,17,18,19] and Aboav laws [19,20,21,22,23,24,25,26,27,28,29,30]. However, the validity of these laws was studied for biological tissues, including cells constituting human muscles [55]. The Aboav law was reported for mitosis in vegetable tissues [31]. It was also shown that the Lewis empirical, linear relationship between the average area of a cell and the number of its sides in two-dimensional mosaics corresponds to maximal arbitrariness in the cellular distribution observed in in epithelial mosaics [20].

## 4. Droplet Clusters and Their Analysis with Voronoi Diagrams.

**Figure 5:**
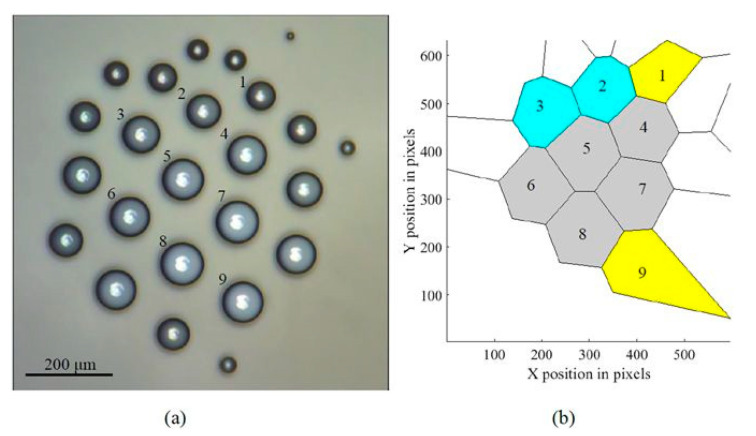
Self-organization of a droplet cluster is demonstrated. (**a**) The image of the cluster and (**b**) the Voronoi tessellation of the cluster. The scale bar is 200 µm. Yellow (1,9), gray (4–8), and blue (3,2) polygons have five, six, and seven neighbors (edges), respectively [56].

Another area of capillary phenomena in which Voronoi diagrams are used is the self-assembled levitating clusters of water microdroplets. Such clusters emerge over locally heated spots of a liquid surface [56,57,58,59,60,61]. Growing and condensing droplets with a typical diameter of 5–100 μm levitate at an equilibrium height [56,57,58,59,60,61]. Their weight is equilibrated by the drag force of the ascending air-vapor jet rising over the heated spot (Figure 5).

Droplets form a monolayer and arrange into a hexagonally ordered structure called a cluster. Due to the attraction to the center of the heated area combined with aerodynamic repulsion between the droplets, the clusters form structures that are quite diverse and different from densest packing of hard spheres [57]. 

**Figure 6:**
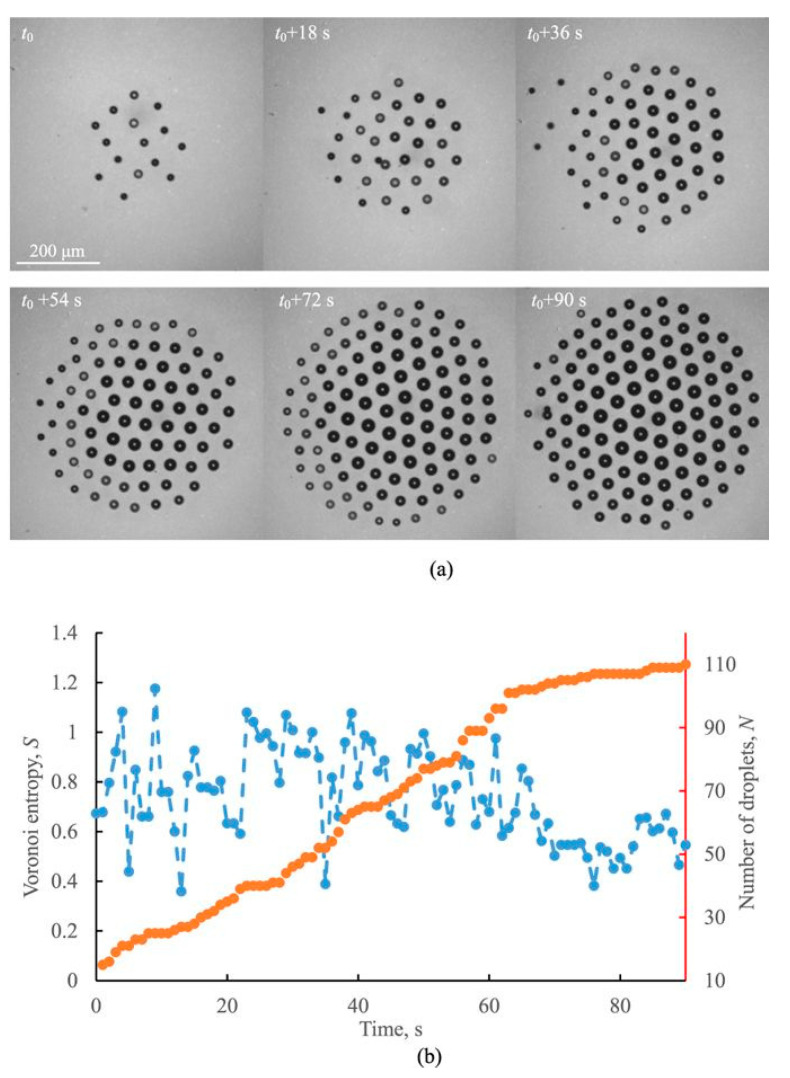
(**a**) Self-assembly of a droplet cluster over a heated water and (**b**) the Voronoi entropy, *S* (blue), correlated with the number of droplets, *N* (red) [56]. The scale bar is 200 µm.

Evolution of a typical growing water cluster is shown in Figure 6. To construct the Voronoi diagram and to calculate the Voronoi entropy, we used the modules of the MATLAB program developed at the Department of Physics and Astronomy at the University of California (Department of Physics and Astronomy University of California). Since the radii of droplets (ca 5–100 µm) were much smaller than the capillary length [4] they kept strictly spherical shape and consequently their centers were considered as the seed points or *nuclei* (see Figure 3).

The Voronoi entropy decreases with increasing time and the number of droplets [56,57]. Newly arriving droplets disturb the hexagonal structure, and the size of the droplets affects the Voronoi entropy. As a result, the Voronoi entropy grows immediately after a new droplet joins the cluster [56]. Following that, the entropy decreases due to the ordering of the cluster arrangement. Most tests also showed a correlation between the entropy and the fraction of hexagonal clusters. This is because the hexagonal arrangement provides the densest 2D packing [57]. Levitating monodisperse microdroplet clusters with 1–28 droplets formed over a locally heated water layer have been reported recently [57]. 

**Figure 7:**
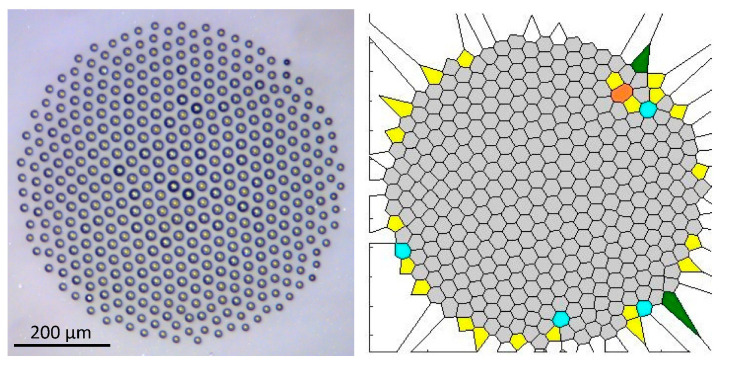
A large droplet cluster and its Voronoi diagram are shown.

**Figure 8:**
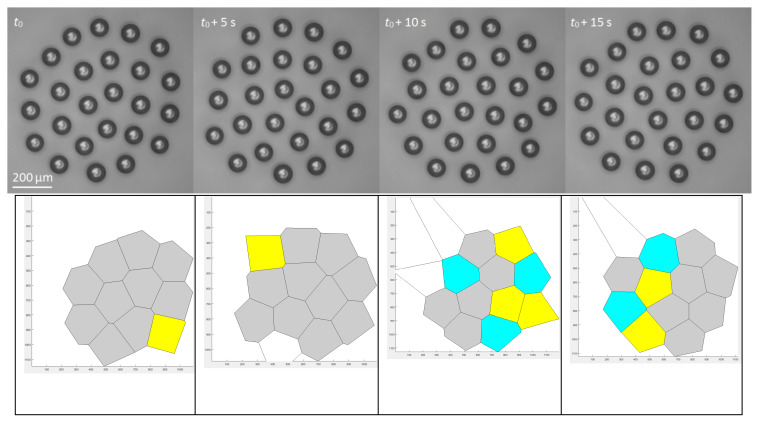
A small droplet cluster during self-assembly and its corresponding Voronoi diagrams are shown [57]. The scale bar is 200 µm. Yellow, gray, and blue polygons have five, six, and seven neighbors (edges), correspondingly.

Figure 7 shows a relatively large droplet cluster and its Voronoi diagram. The structure of the cluster is ordered at the center, while there are defects at the periphery. The value of the Voronoi entropy is *S_vor_* = 0.335. Figure 8 depicts the self-assembly stages of a small droplet cluster [56].

Calculation of the dynamic Voronoi entropy enabled not only quantification of ordering on droplet clusters, but also characterization of its temporal evolution [56,57]. 3D Voronoi analysis enabled quantification the clustering of inertial particles in homogeneous isotropic turbulence using data sets, extracted from experiments performed with microbubbles [62]. Voronoi analysis also allowed distinguishing the clustering behavior of heavy, neutrally buoyant, and light particles in turbulent flows [62].

## 5. The Relation between Voronoi Entropy and Thermodynamic Entropy 

The complicated relation between the Voronoi entropy and thermodynamic entropy was extensively addressed in Reference [10]. The relationship between the precisely defined Voronoi free volume information entropy and the thermodynamic entropy was established for the 3D hard-disk and hard-sphere systems [10]. The Voronoi free volume of any hard particle was defined as the difference between its Voronoi volume and the minimal cell volume occurring at a regular close packing. It was demonstrated that the maximal entropy formalism, when applied to the free volume entropy, predicts an exponential distribution which approaches disorder in the dense random-packing limit [10]. The obvious difference between the 2D Voronoi entropy and thermodynamic entropy should be mentioned: (1) the 2D Voronoi entropy is an intensive property of the pattern (in other words the Voronoi entropy of the given pattern does not grow with the growth of the pattern); whereas the thermodynamic entropy is an extensive thermodynamic value, and it is increased with the growth of the system; (2) the thermodynamic entropy is the relativistic invariant of the system [63,64], whereas the Voronoi entropy is not. More investigations in the field are desirable. 

## 6. Conclusions

Several methods can be used to quantify the orderliness of 2D patterns: The Minkowski functionals [45], Fourier analysis [44], and correlation functions [47]. An alternative method is the calculation of the entropy of the Voronoi diagram, which is the 2D analogy of 3D Wigner–Seitz partition [65,66]. The diagram itself traces back to Johannes Kepler and Rene Descartes [6,7]. The method was revived by Dirichlet [9] and Voronoi [5] and became popular for quantitative characterization of 2D and 3D patterns. This approach has been successfully applied to the characterization of surface self-assembly of biological and natural mosaics, occurring on the broad diversity of spatial scales (from molecular to macroscopic ones). The Voronoi analysis is also effective for the analysis of surface porous structures and droplet clusters [56,57,58,59,60,61], enabling in situ characterization of ordering. We conclude that the use of Voronoi diagrams is a powerful tool enabling analysis and quantification of ordering in a diversity of synthetic and biological systems. The comparison of the effectiveness of Voronoi diagrams for the analysis of 2D ordering vs. Minkowski functionals and Fourier analysis remains an open problem. The relation between the Voronoi entropy and thermodynamic entropy remains unclear and calls for future investigations. 

## References

[1] Widawski G., Rawiso M., Francois B. (1994). Self-organized honeycomb morphology of star-polymer polystyrene films. Nature.

[2] Pitois J., Francois B. (1999). Formation of ordered micro-porous membranes. Eur. Phys. J. B.

[3] Karthaus O., Cieren X., Shimomura M., Hasegawa T. (2000). Water-assisted formation of micrometer-size honeycomb patterns of polymers. Langmuir.

[4] Bormashenko E. (2017). Breath-figure Self-assembly, a Versatile Method of manufacturing membranes and porous structures: physical, chemical and technological aspects. Membranes.

[5] Voronoi G. (1908). Nouvelles applications des paramètres continus à la théorie des formes quadratiques. Deuxième mémoire. Recherches sur les paralléloèdres primitifs. Reine Angew. Math..

[6] Descartes R. (1644). Principia Philosophiae.

[7] Liebling T.M., Pournin L. (2012). Voronoi diagrams and Delaunay triangulations: Ubiquitous Siamese Twins. Doc. Math..

[8] Snow J. (1855). Report on the Cholera Outbreak in the Parish of St. James, Westminster: during the autumn of 1854.

[9] Dirichlet G.L. (1850). Über die Reduction der positiven quadratischen Formen mit drei unbestimmten ganzen Zahlen. J. Reine Angew. Math..

[10] Kumar V.S., Kumaran V. (2005). Voronoi cell volume distribution and configurational entropy of hard-spheres. J. Chem. Phys..

[11] Barthélemy M. (2011). Spatial networks. Phys. Rep..

[12] Weaire D., Rivier N. (1984). Soap, cells and statistics—random patterns in two dimensions. Contemporary Phys..

[13] Blatov V.A. (2004). Voronoi–Dirichlet polyhedra in crystal chemistry: theory and applications. Crystallography Reviews.

[14] Limaye A.V., Narhe R.D., Dhote A.M., Ogale S.B. (1996). Evidence for convective effects in breath figure formation on volatile fluid surfaces. Phys. Rev. Lett..

[15] Lewis E.T. (1928). The correlation between cell division and the shapes and sizes of prismatic cell in the epidermis of Cucumis. Anat. Rec..

[16] Lewis F.T. (1930). A volumetric study of growth and cell division in two types of epithelium-the longitudinally prismatic cells of Tradescantia and the radially prismatic epidermal cells of Cucumis. Anat. Rec..

[17] Lewis E.T. (1943). The geometry of growth and cell division in epithelial mosaics. Am. J. Bot..

[18] Lewis F.T. (1944). The geometry of growth and cell division in columnar parenchyma. Am. J Bot..

[19] Chiu S.N. (1995). Aboav-Weaire’s and Lewis’ laws—A review. Mater. Charact..

[20] Rivier N., Lissowski A. (1982). On the correlation between sizes and shapes of cells in epithelial mosaics. J. Phys. A Math. Gen..

[21] Sánchez-Gutiérrez D., Tozluoglu M., Barry J.D., Pascual A., Mao Y., Escudero L.M. (2016). Fundamental physical cellular constraints drive self-organization of tissues. EMBO J..

[22] Saraiva J., Pina P., Bandeira L., Antunes J. (2009). Polygonal networks on the surface of Mars; applicability of Lewis, Desch and Aboav–Weaire laws. Phil. Mag. Lett..

[23] Steyer A., Guenoun P., Beysens D., Knobler C.M. (1990). Two-dimensional ordering during droplet growth on a liquid surfaceth on a liquid surface. Phys Rev. B.

[24] Pietsch T., Gindy N., Fahmi A. (2009). Nano- and micro-sized honeycomb patterns through hierarchical self-assembly of metal-loaded diblock copolymer vesicles. Soft Matter.

[25] Park M.S., Kim J.K. (2004). Breath figure patterns prepared by spin coating in a dry environment. Langmuir.

[26] Bormashenko E., Musin A., Whyman G., Barkay Z., Zinigrad M. (2013). Revisiting the fine structure of the triple line. Langmuir.

[27] Madej W., Budkowski A., Raczkowska J., Rysz J. (2008). Breath figures in polymer and polymer blend films spin-coated in dry and humid ambience. Langmuir.

[28] Rivier N. (1985). Statistical crystallography structure of random cellular networks. Phil. Mag.B.

[29] Aboav D.A. (1970). The arrangement of grains in a polycrystal. Metallography.

[30] Weaire D. (1974). Some remarks on the arrangement of grains in a polycrystal. Metallography.

[31] Mombach J.C.M., de Almeida R.M.C., Iglesias J.R. (1993). Mitosis and growth in biological tissues. Phys.Rev. E.

[32] Jarai-Szabo F., Zoltan N. (2007). On the size distribution of Poisson Voronoi cells. Phys. A.

[33] Zhu H.X., Thorpe S.M., Windle A.H. (2001). The geometrical properties of irregular two-dimensional. Phil. Mag. A.

[34] Shirriff K. (1998). Generating fractals from Voronoi diagrams. Comput. Graph..

[35] Delaunay B. (1934). Sur la sphère vide. Bulletin de l’Académie des Sciences de l’URSS, Classe des Sciences Mathématiques et Naturelles.

[36] Sung B.J., Yethiraj A. (2010). Structure of void space in polymer solutions. Phys Rev E Stat Nonlin. Soft Matter Phys..

[37] Danielsson M., Parks D.M., Boyce M.C. (2002). Three-dimensional micromechanical modeling of voided polymeric materials. J. Mech. Phys. Solids.

[38] Bigioni T.P., Lin X.M., Nguyen T.T., Corwin E.I., Witten T.A., Jaeger H.M. (2006). Kinetically driven self assembly of highly ordered nanoparticle monolayers. Nat. Mater..

[39] Yun S.-H., Yoo S., Jung J.C., Zin W.-C., Sohn B.-H. (2006). Highly Ordered Arrays of Nanoparticles in Large Areas from Diblock Copolymer Micelles in Hexagonal Self-Assembly. Chem. Mater..

[40] Arora H., Du P., Tan K.W., Hyun J.K., Grazul J., Xin H.L., Muller D.A. (2010). Block Copolymer Self-Assembly–Directed Single-Crystal Homo- and Heteroepitaxial Nanostructures. Science.

[41] Xu J., Russell T.P., Ocko B.M., Checco A. (2011). Block copolymer self-assembly in chemically patterned squares. Soft Matter..

[42] Zámbó D., Suzuno K., Pothorszk S., Bárdfalvy D., Holló G., Nakanishi H., Wang D., Ueyama D., Deák A., Lagz I. (2016). Self-assembly of like-charged nanoparticles into Voronoi diagrams. Phys. Chem. Chem. Phys..

[43] Martin C.P., Blunt M.O., Pauliac-Vaujour E., Stannard A., Moriarty P., Vancea I., Thiele U. (2007). Controlling Pattern Formation in Nanoparticle Assemblies via Directed Solvent Dewetting. Phys. Lett..

[44] Lim J.S. (1990). Two Dimensional Signal and Image Processing.

[45] Parker J., Sherman E., van de Raa M., van der Meer D., Samelson L.E., Losert W. (2013). Automatic sorting of point pattern sets using Minkowski functionals. Phys. Rev. E.

[46] Mantz H., Jacobs K., Mecke K. (2008). Utilizing Minkowski functionals for image analysis: A marching square algorithm. J. Stat. Mech. Theor. Exp..

[47] Bormashenko E., Malkin A., Musin A. (2008). Mesoscopic patterning in evaporated Polymer solutions: Poly (ethylene glycol) and room-temperature-vulcanized Polyorganosilanes/-siloxanes Promote formation of honeycomb structures. Macromol. Chem. Phys..

[48] Aitken J. (1895). Breath Figures. Proc. R. Soc. Edinb..

[49] Aitkek J. (1911). Breath figures. Nature.

[50] Rayleigh L. (1911). Breath figures. Nature.

[51] Rayleigh L. (1912). Breath figures. Nature.

[52] Bormashenko E., Pogreb R., Stanevsky O., Bormashenko Y., Stein T., Gendelman O. (2005). Mesoscopic patterning in evaporated polymer solutions: new experimental data and physical mechanisms. Langmuir.

[53] Bormashenko E., Pogreb R., Musin A., Stanevsky O., Bormashenko Y., Whyman G., Gendelman O., Barkay Z. (2006). Self-assembly in evaporated polymer solutions: Influence of the solution concentration. J. Colloid Interface Sci..

[54] Alinchenko M.G., Anikeenko A.V., Medvedev N.N., Voloshin V.P., Mezei M., Jedlovszky P. (2004). Morphology of Voids in Molecular Systems. A Voronoi-Delaunay analysis of a simulated DMPC membrane. J. Phys. Chem. B.

[55] Sánchez-Gutiérrez D., Sáez A., Gómez-Gálvez P., Paradas C., Escudero L.M. (2017). Rules of tissue packing involving different cell types: human muscle organization. Sci. Rep..

[56] Fedorets A.A., Frenkel M., Shulzinger E., Dombrovsky L.A., Bormashenko E., Nosonovsky M. (2017). Self-assembled levitating clusters of water droplets: pattern-formation and stability. Sci. Rep..

[57] Fedorets A.A., Frenkel M., Bormashenko E., Nosonovsky M. (2017). Small levitating ordered droplet clusters: stability, symmetry, and Voronoi Entropy. J. Phys. Chem. Lett..

[58] Fedorets A.A. (2004). Droplet cluster. JETP Lett..

[59] Fedorets A.A. (2005). On the Mechanism of non-coalescence in a droplet cluster. JETP.

[60] Fedorets A.A. (2012). Mechanism of stabilization of location of a droplet cluster above the liquid–gas interface. Tech. Phys. Lett..

[61] Fedorets A.A., Dombrovsky L.A. (2017). Generation of levitating droplet clusters above the locally heated water surface: A thermal analysis of modified installation. Int. J. Heat Mass Transf..

[62] Tagawa Y., Mercado J.M., Prakash V.N., Calzavarini E., Sun C., Lohse D. (2012). Three-dimensional Lagrangian Voronoi analysis for clustering of particles and bubbles in turbulence. J. Fluid Mech..

[63] Tolman R.C. (1934). Relativity, Themodynamics and Cosmology.

[64] Bormashenko E. (2007). Entropy of relativistic mono-atomic gas and temperature relativistic transformation in thermodynamics. Entropy.

[65] Wigner E., Seitz F. (1933). On the Constitution of metallic Sodium. Phys. Rev..

[66] Ashcroft N.W., Mermin N.D. (1976). Solid State Physics.

